# Tetraploid Wheat Landraces in the Mediterranean Basin: Taxonomy, Evolution and Genetic Diversity

**DOI:** 10.1371/journal.pone.0037063

**Published:** 2012-05-16

**Authors:** Hugo R. Oliveira, Michael G. Campana, Huw Jones, Harriet V. Hunt, Fiona Leigh, David I. Redhouse, Diane L. Lister, Martin K. Jones

**Affiliations:** 1 Department of Archaeology, University of Cambridge, Cambridge, United Kingdom; 2 Department of Human Evolution Biology, Harvard University, Cambridge, Massachusetts, United States of America; 3 National Institute for Agricultural Botany, Cambridge, United Kingdom; 4 McDonald Institute for Archaeological Research, Cambridge, United Kingdom; The Australian National University, Australia

## Abstract

The geographic distribution of genetic diversity and the population structure of tetraploid wheat landraces in the Mediterranean basin has received relatively little attention. This is complicated by the lack of consensus concerning the taxonomy of tetraploid wheats and by unresolved questions regarding the domestication and spread of naked wheats. These knowledge gaps hinder crop diversity conservation efforts and plant breeding programmes. We investigated genetic diversity and population structure in tetraploid wheats (wild emmer, emmer, rivet and durum) using nuclear and chloroplast simple sequence repeats, functional variations and insertion site-based polymorphisms. Emmer and wild emmer constitute a genetically distinct population from durum and rivet, the latter seeming to share a common gene pool. Our population structure and genetic diversity data suggest a dynamic history of introduction and extinction of genotypes in the Mediterranean fields.

## Introduction

Tetraploid wheats have played a critical role in human history. Durum is the primary wheat for pasta and semolina production and the second most cultivated wheat after bread wheat (*Triticum aestivum* L.). Although a ‘relic’ crop today, emmer wheat is used for bread making, animal feed and as a genetic resource for the improvement of durum and bread wheat varieties [1]. Rivet or cone wheat is also a naked wheat variety with a broader cultivation tolerance range than durum, being grown in northern latitudes such as the UK [2]. Rivet practically disappeared from cultivation during the 20^th^ century and its extinction was prevented only by inclusion of rivet accessions in germplasm bank collections [3]. Although many studies have focused on the details of wheat domestication process, few have considered its subsequent evolution and history and the processes underlying the formation of different landraces (traditional varieties lacking formal crop improvement, adapted to the local environments of the particular locality where they are grown and associated with small-scale farming). [4].

Tetraploid wheats are genetically and morphologically diverse and their evolution under domestication has not been fully elucidated [5]. Genetic and archaeological evidence indicates that cultivated emmer evolved from the tetraploid wild emmer in the Fertile Crescent around the 8^th^ millennium BCE [5]–[6]. Free-threshing tetraploid wheats (durum and rivet) appear in the archaeological record shortly after emmer in the Near East and it has been assumed they evolved from previously domesticated emmer stands [6]–[7]. Based on restriction fragment length polymorphism data, Luo and colleagues [8] proposed that durum wheat evolved after emmer in the eastern Mediterranean. Due to the difficulty of distinguishing between free-threshing wheats (tetraploid durum and rivet, and hexaploid bread wheat) in the archaeological record, the history of these crops is still unclear. Nevertheless, bread wheat (*T. aestivum* L.) has been demonstrated to have evolved from a domesticated tetraploid, most likely emmer, and the D-genome donor *Aegilops tauschii* [9]. Both hulled and free-threshing forms were part of the initial crop package introduced into Europe and North Africa during the Neolithic but adoption of these crops was not uniform throughout the Mediterranean Basin. Free-threshing wheats were preferred in the western Mediterranean basin since the start of agriculture in these regions [10], whereas emmer was the staple crop in Ancient Egypt until the introduction of durum in the Hellenistic Period [11]. Some emmer landraces have been proposed to be more-or-less established in their associated localities since their initial introduction in the Neolithic, therefore having the potential to be informative about the process of agricultural spread into these regions [12].

A rigorous taxonomical classification for wheat species is important for the conservation of wheat biodiversity [13]. Imprecise knowledge of tetraploid wheats' evolutionary histories, the lack of a universally accepted definition of “landrace” and the absence of unified protocols for sampling and storage of landraces could result in significant losses of genetic diversity and undermine both in-situ and ex-situ conservation efforts [14]–[15]. Despite extensive research, the taxonomy of tetraploid wheats, including the wild emmer and cultivated forms, remains a source of contention. Several classification systems have been proposed based on morphological, cytological or genetic characters [16]. Both Van Slageren [17] and MacKey [18] consider all forms as subspecies of *Triticum turgidum* (for example in the Van Slageren system durum is *T. turgidum* subsp. *durum*). MacKey further classifies durum and rivet as convarieties of the subspecies *T. turgidum* subsp. *turgidum* (*T. turgidum* subsp. *turgidum* conv. *durum*). Conversely, Dorofeev and colleagues [19] classify all tetraploid wheat forms as individual species (*T. durum*). Previous genetic studies based on expressed genes in the nuclear and chloroplast genomes have focused on the origin of the different genomes and the relationship between domesticated and wild wheats [8], [13], [20]. Previous genetic studies based on simple sequence repeats (SSRs) have sought to quantify genetic diversity [21]–[23], to investigate population structure [24] and only seldom to investigate phylogenetic relationships within the whole Triticeae tribe [25].

We analysed four classes of genetic markers: nuclear simple-sequence repeats (nuSSRs), chloroplast SSRs (cpSSRs), insertion site-based polymorphisms (ISBPs) and functional markers in expressed genes to investigate within landrace and between landrace genetic diversity in the Mediterranean Basin. We addressed the following questions: 1) how genetically diverse are tetraploid wheat landraces?; 2) can the genetic diversity in neutral markers elucidate the taxonomy of tetraploid wheats?; 3) can the geographic distribution of genetic diversity reveal the evolution and history of tetraploid wheats in the Mediterranean basin?

## Materials and Methods

### Plant Material

For *within landrace* genetic diversity analysis, 75 individuals from seven tetraploid wheat landrace accessions from the Iberian Peninsula were analysed (Table 1). These accessions included two durums (Candeal Grão Escuro and Recio), which had been conserved *ex-situ* in germplasm banks, and five emmers (Tios A, Tios B, Zureda, Conforcos and Pelugano) preserved by cultivation *in situ* in Asturias, Spain. Population structure in the latter accessions was previously studied using chloroplast and nuclear SSRs [26]. DNA was extracted from individual young seedlings using the Tanksley method [27].

**Table 1:** Measures of within-landrace genetic diversity in seven tetraploid wheat accessions.

Landrace	No. Genotypes	Freq. Genotypes	Hz	GD	PIC	Rare Alleles	Hg (%)	Null Alleles (%)
CGE	2	15/1	0.067	0.042	0.033	1 (300)	0.34	0.67
Recio	2	19/1	0.067	0.039	0.031	1 (240)	0.42	0
Tios A	2	6/2	0	0.094	0.088	2 (120)	1.67	3.4
Tios B	1	8	0	0.081	0.080	0 (120)	0	2.5
Zureda	1	7	0	0.085	0.083	0 (105)	0	3.81
Conforcos	4	3/2/2/1	0	0.360	0.140	7 (120)	5.84	1.67
Pelugano	6	3/1/1/1/1/1	0	0.360	0.300	28 (120)	23.3	5
**Mean**	**2.5**	**-**	**0.019**	**0.152**	**0.108**	**5,6 (161)**	**5**	**2**

No. Genotypes: number of different genotypes detected in the sample; Freq. Genotypes: number of individual plants with a particular genotype within the sample; Hz: Heterozygosity; GD: Gene Diversity; PIC: polymorphism information content; M/P markers: number of polymorphic/monomorphic markers; Rare Alleles: number of rare alleles (an allele other than the most frequent one for each loci analysed) detected in the landrace (the total number of alleles detected for each landrace accession is under brackets); Hg (%): heterogeneity within varieties calculated as the number of alleles, other than the most frequent one, detected for a particular marker/landrace combination, considering the totality of alleles genotyped. e.g. for Recio, given 20 individuals analysed at 15 SSR loci, there is 1 instances of a rare allele being detected, so heterogeneity is 0.34%.

For *between landrace* genetic diversity analysis, 244 accessions were assayed (Table S1). These included 20 wild emmer, 20 emmer, 21 rivet and 174 durum accessions from the Mediterranean basin. Also included were 9 hexaploid *T. aestivum* landrace accessions as an out-group. Two cultivars, *T. aestivum* var. Chinese Spring and *T. turgidum durum* var. Langdon, were used as control standards on every plate analysed. DNA was extracted from a bulk of 6–8 young seedlings per accession using a modified Tanksley method [27]. Extracted DNA was quantified using a NanoDrop™ 1000 spectrophotometer and all extracts were diluted to a final concentration of 50 ng µL^−1^.

### Nuclear and Chloroplast SSR Markers

A panel of 37 nuSSRs, including genomic [28] and EST-derived SSRs [29], was created in order to sample all chromosomes in the A and B genomes (Table S2; Details S1). Twenty-nine markers that produced reliable genotyping profiles were selected for subsequent analysis. In order to contrast bi-parentally inherited nuSSRs with maternally inherited markers, we also genotyped the accessions with five cpSSRs [30]. Chloroplast haplotypes were created by combining the alleles observed for the set of five cpSSR loci in each accession. The geographic distribution of these chloroplast haplotypes was mapped. Forward primers for nuSSRs were designed with an extra M13-tail at the 5′ end for attachment to a universal fluorescently-labelled M13 primer [31]. The forward primer of the EST-SSR and cpSSR markers was labelled with one of four fluorescent dyes: 6-FAM, PET, NED or VIC. All PCR amplification products were visualised on 2% TBE-agarose gels stained with ethidium bromide. SSR PCR products were electrophoresed on an ABI PRISM® 3730 DNA Analyser and chromatograms were analysed with GeneMapper® ver.3.7 software. Alleles were scored using the binning function. A single allele was scored for each marker in each accession corresponding to the strongest peak. A subset of 15 nuSSRs was analysed to calculate *within landrace* genetic diversity (Table S3), while the complete panel was analysed to investigate *between landrace* genetic diversity.

### Functional Markers and ISBPs

To contrast with the population structure based on putatively neutral SSRs we investigated allelic states of functional markers in genes related to the environmental response of the plants, and thus putatively under natural selection. We used a PCR-based system to detect deletions in the *Ppd-A1* gene in chromosome 2A of tetraploid wheats [32]; if a variety does not have the deletion it is photoperiod sensitive, flowering in response to the onset of longer days in spring, and the presence of a band with 452 bp is expected in the gel electrophoresis. If the GS-100 or the GS-105 types of deletions are present, 380 bp and 290 bp bands are expected respectively and the varieties are expected to be photoperiod insensitive, flowering earlier then sensitive varieties. The panel of accessions was also screened for the presence/absence of a deletion in the intron 1 of the *VRN-A1* gene, involved in the vernalisation response and spring/winter growth habit in wheat [33]. We also typed four ISBP (insertion-site based polymorphism) retrotransposon-based markers on chromosome 3B [34]. ISBP markers were scored by the presence/absence of four diagnostic bands (one for each marker) on an agarose gel. For each ISBP marker, the most replicable band was selected as diagnostic. ISBP haplotypes were determined for each accession by combining the alleles observed for the four loci. See Details S1 for PCR details.

### Genetic Structure

We clustered landraces into populations based on the nuclear SSR data without any *a priori* information such as subspecies or geographical provenance using STRUCTURE 2.2 [35]–[36]. Models assuming one (*K* = 1) to ten (*K* = 10) clusters were tested using 1,000,000 MCMCs and 500,000 burn-in runs, with the admixture model. Twenty replicate runs were performed for each value of *K*. The best-fit model was determined by plotting the natural log probability of the data against *K* [37], by determining Δ*K* [38] and by checking for consistency between *Q-*matrices from replicate runs in each value of *K* using the program CorrSieve 1.1 [39]. For subsequent analysis, individual accessions were assigned to the population with the highest proportional membership. A landrace was considered mixed when proportional membership to any single population was less than 60%. Clusters were mapped in ArcGIS 9.0 (ESRA) and using the Generic Mapping Tools Software package (http://gmt.soest.hawaii.edu).

Genetic structure was further investigated on the 244 accessions by means of principal component analysis (PCA) based on microsatellite allele frequency data from the 29 nuSSR panel using the R packages ade4 [40] and adegenet [41].

### Genetic Diversity and Genetic Distance

Gene diversity, polymorphic information content (PIC) and allele richness for nuSSRs were calculated using PowerMarker [42] in each of the populations defined by STRUCTURE. Genetic diversity was estimated for all markers. Pairwise genetic distances between individual accessions were calculated in PowerMarker based on nuclear SSR data using the *D_C_* genetic distance measure [43], an appropriate method for microsatellite data [44]. *D_C_* genetic distances were also calculated separately for the sets of microsatellites located in the A and B genomes. Finally, we grouped our landraces in eight *a priori* defined populations: emmer accessions, rivet accessions, and durum accessions grouped by regional geographical provenance i.e. Southwest Asia, Southeast Europe, Central Mediterranean, Southwest Europe, Northwest Africa and Northeast Africa (Table 2). *D_C_* genetic distances between these a priori populations were calculated, and dendrograms were constructed in PowerMarker using a neighbour-joining clustering method with bootstrap support (1000 replicates) obtained by re-sampling the allelic frequency data. A majority-rule consensus tree was produced using the CONSENSUS routine in the PHYLIP package available in the Mobyle portal (http://mobyle.pasteur.fr/cgi-bin/portal.py) and subsequently manipulated in Dendroscope version 2.5 [45].

**Table 2:** Genetic diversities in *a priori* defined populations of tetraploid wheats.

Population	Sample Size	Mean Alleles per Locus	Unique Alleles	Gene Diversity	PIC
Wild emmer	20	9.8	111	0.833	0.820
Emmer	20	6.7	21	0.708	0.678
Rivet	21	5.8	3	0.682	0.643
Durum SW Europe	62	7.5	12	0.671	0.632
Durum SWAsia	28	6.0	2	0.631	0.595
Durum SE Europe	12	4.5	2	0.635	0.592
Durum Central Mediterranean	15	4.6	1	0.603	0.558
Durum NE Africa	17	4.7	2	0.575	0.535
Durum NWAfrica	40	5.7	6	0.546	0.504
Bread wheat	9	4.5	22	0.652	0.616

Bread wheat (*Triticum aestivum*) is included for comparison. Measures are based on 29 nucSSRs. Regions and countries are as follows: Southwest Asia (Turkey, Lebanon, Israel, Syria, Iran, Iraq), Southeast Europe (Greece, Cyprus, Macedonia), Central Mediterranean (France, Italy, Malta, Croatia), Southwest Europe (Portugal, Spain), Northwest Africa (Morocco, Algeria, Tunisia) and Northeast Africa (Libya, Egypt).

### Association between Populations and Geographic/Climatic Factors

To investigate the effect of geographic distance and environmental factors on the distribution of genetic diversity in tetraploid wheats, we studied a subset of 53 Iberian durum accessions for which a precise geographic location was reported in the passport data. Great circle distances were calculated for each pair of individual landraces. For each known landrace location a series of environmental parameters were obtained using ArcGIS (ESRA) and the WorldClim database (www.worldclim.org). These included altitude (*r*), mean annual temperature (*s*), maximum temperature of the warmest month (*t*), minimum temperature of the coldest month (*u*), mean annual precipitation (*v*), precipitation of the driest month (*w*) and precipitation of the wettest month (*x*). Environmental distances (*D_ENVij_*) between two locations *i* and *j* were calculated using equation 1 (adapted from [46]). Correlations between *D_C_*, great circle and log-transformed *D_ENVij_* distances were investigated using Mantel tests (999 iterations per test: [47]) in PopTools [48].
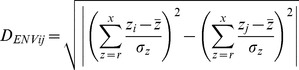
(1)


## Results

### Genetic Diversity within Landraces

58 alleles (total) were detected in the 75 individual plants analysed with 15 nuSSRs. The number of alleles detected per locus ranged between 2 (*Xgwm540*) and 6 (*Xgwm4*6) with a mean of 4 alleles per locus (Table S3). PICs ranged between 0.372 (*Xgwm169*) and 0.776 (*Xgwm160* and *Xgwm60*) with a mean of 0.622 (Table S3). Heterozygosity was observed only for one marker—all individuals genotyped in accessions Recio and Candeal Grão Escuro had two peaks for marker *Xgwm160*. It is uncertain if this is due to real heterozygosity or to a PCR artefact.

Although we observed variable degrees of within-landrace genetic diversity, heterogeneity within landraces was low (the mean number of alleles per locus within individual accessions = 1.2) (Table 1). The *in situ* conserved Conforcos and Pelugano accessions were more diverse than the other accessions with 4 and 6 different genotypes being detected, respectively, and 3 and 9 markers out of the total of 15 being polymorphic within the accession, resulting in heterogeneity levels of 5.84% and 23.3% respectively (Table 1).

### Genetic Diversity between Landraces

498 SSR alleles (including both nuSSRs and cpSSRs) were detected in the panel of tetraploid wheat accessions (excluding the *T. aestivum* outgroup). The number of alleles per locus ranged between 4 (*WCt15*) and 33 (*Xgwm*6) with a mean of 14.6 alleles per locus (Table S4). Null alleles were observed for all marker types. These were treated as ‘missing data’ and omitted from subsequent analysis. Interestingly, marker *Xgwm169* null alleles occurred in emmer landraces from fields in the Asturias region only, but produced quality amplification products in all other accessions, including emmer from other locations. Conversely, *Xgwm46* failed to produce an amplification product in all tested emmer accessions except those from Spain.

**Figure 1:**
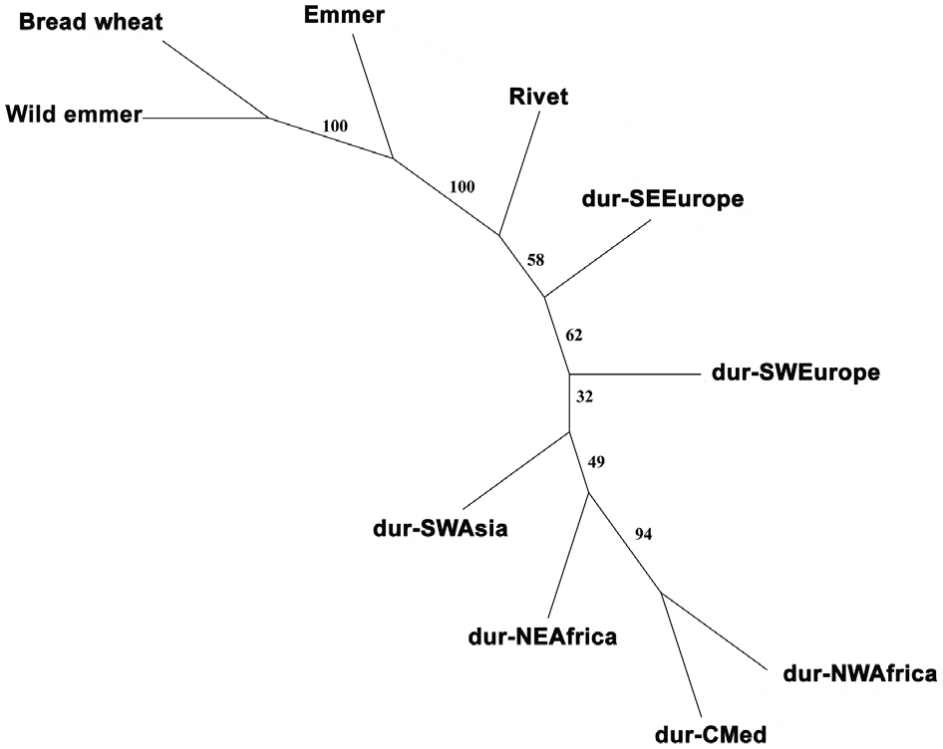
Neighbour-joining tree between *a priori* defined tetraploid wheat populations. The tree was constructed from *D_C_* genetic distances using wild emmer to root the tree and 100 bootstrap replicates.

Wild emmer was the most diverse of all ten *a priori* defined populations (gene diversity of 0.833 and 111 unique alleles) followed by emmer (gene diversity of 0.708) and rivet (gene diversity of 0.682), while Northwest African durum was the least diverse (gene diversity of 0.546) (Table 2). Among durum populations, the highest gene diversity was found in Southwest Europe (0.669). Within the *a priori* populations, emmer is genetically closer to wild emmer than to any of the other groups (Table 3). Interestingly, the durum groups more closely related to emmer are from Southwest and Southeast Europe. Rivet is genetically closer to all durum groups than to emmer, wild emmer or bread wheat. Durums from Northwest Africa (Maghreb) are closer to the durums from Central Mediterranean (Italy, France and Croatia). In the consensus NJ tree, durum groups were grouped in different branches from wild emmer, emmer and bread wheat, with rivet in between them (Figure 1). Southeast Europe was the geographic area with durum accessions genetically closer to emmer. As in the genetic distance matrix, durums from Northwest Africa (Maghreb) clustered more closely with those from the Central Mediterranean than with those from Southwest Europe (Iberia), in contrast to the results previously reported by Moragues et al [49]. Pairwise genetic distance matrices between all accessions, considering the A and B genome markers separately, were weakly correlated (*r = *0.425, *p*<0.001), indicating that the phylogenetic information contained in the A and B genomes is different.

**Table 3:** Genetic distances between *a priori* defined populations of tetraploid wheats.

	Bread Wheat	dCM	dNEA	dNWA	dSEE	dSWA	dSWE	Emmer	Rivet	Wild Emmer
**Bread Wheat**	0	-	-	-	-	-	-	-	-	-
**dCM**	0.240	0	-	-	-	-	-	-	-	-
**dNEA**	0.271	0.083	0	-	-	-	-	-	-	-
**dNWA**	0.279	0.043	0.079	0	-	-	-	-	-	-
**dSEE**	0.235	0.093	0.122	0.114	0	-	-	-	-	-
**dSWA**	0.237	0.092	0.083	0.091	0.093	0	-	-	-	-
**dSWE**	0.219	0.051	0.059	0.047	0.060	0.059	0	-	-	-
**Emmer**	0.188	0.196	0.203	0.232	0.170	0.174	0.160	0	-	-
**Rivet**	0.204	0.092	0.099	0.107	0.068	0.087	0.043	0.152	0	-
**Wild Emmer**	0.145	0.192	0.223	0.230	0.174	0.194	0.162	0.144	0.157	0

Bread wheat (*Triticum aestivum*) is included for comparison. Distances are based on 29 nuSSRs. dCM: durum Central Mediterranean; dNEA: durum Northeast Africa; dNWA: durum Northwest Africa; dSEE: durum Southeast Europe; dSWA: durum Southwest Asia; dSWE: durum Southwest Europe.

### Population Structure

STRUCTURE runs including all the tetraploid wheat accessions and nine *T. aestivum* landraces found well-supported population structure among wheats with a degree of admixture between clusters. In all models, most accessions were indicated to have received alleles from more than a single group. Analyses of *ΔK*, *LnP(D)* and *Q*-matrix correlations indicated that the solutions *K* = 2 and *K* = 6 were stable and well-supported (Figure S1). The sub-populations identified in the *K* = 6 model were further confirmed by running STRUCTURE under the same modelling conditions with subsets of accessions (Figure S2; Figure S3; Figure S4). We therefore considered models *K* = 2 and *K* = 6 for further analysis.

**Figure 2:**
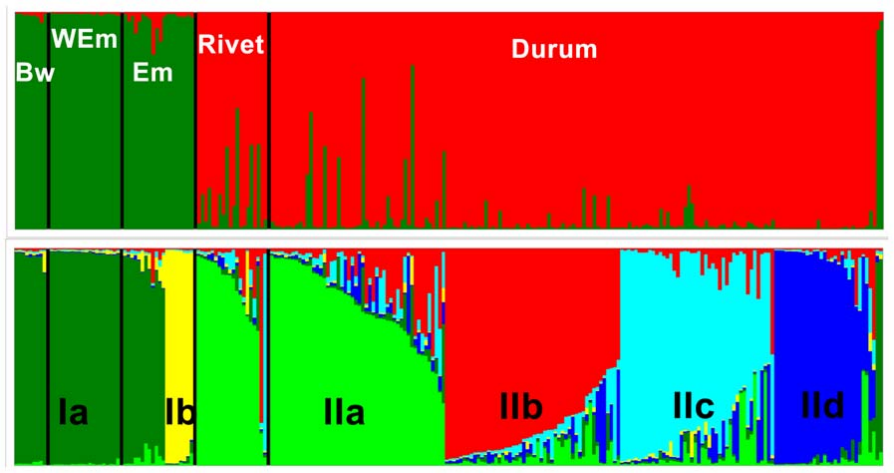
Clustering of 244 wheat accessions based on multilocus genotype analysis using STRUCTURE. Each accession is depicted by a vertical line segmented into *K* coloured sections. The length of each section is proportional to the estimated membership coefficient (*Q*) of the individual accession to each one of the *K* clusters. The black vertical lines are separators between the different forms of wheat (BW: bread wheat; WEm: wild emmer; Em: emmer; Rivet and Durum). The upper panel depicts a model with two clusters (*K* = 2) and the lower panel a model with six clusters (*K* = 6). The black labels in the latter indicate the groups identified.

In the *K* = 2 model, two accessions could not be assigned to either population with a proportion of at least 60%.The remainder were divided into two groups: Group I (dark green), containing wild emmer, emmer and bread wheat, and Group II (red), including rivet and durum accessions (Figure 2).

**Figure 3:**
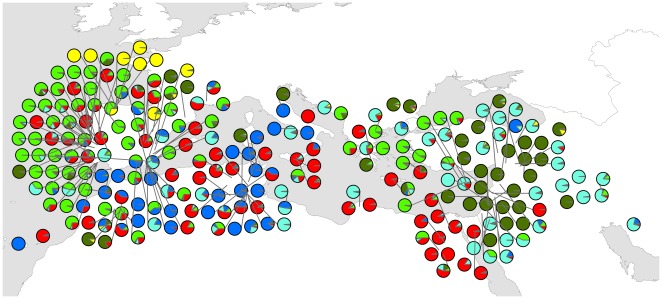
Geographical distribution of tetraploid wheat clusters. Cluster membership was determined using STRUCTURE assuming six clusters (*K* = 6). Each accession is depicted as a pie chart with the proportional membership of its alleles to each one of the six groups: group Ia (dark green), group Ib (yellow), group IIa (light green), group IIb (red), group IIc (light blue) and group IId (dark blue).

In the *K* = 6 model, nine accessions were considered admixed using the 60% assignment criterion. This model separates a group of emmer landraces (n = 8) from Spain (Group Ib – yellow) from the remainder of Group I (Group Ia – dark green) (Figure 2, Figure 3). Group II is divided into 4 clusters (IIa - d) (Figure 2). Group IIa (light green) includes 47 accessions of durum and rivet from the Iberia Peninsula, 11 accessions from Southeast Europe and 4 from Morocco (Figure 3). Group IIb (red) includes 49 durum and one rivet accessions distributed throughout the Mediterranean except in Southwest Asia (Figure 3). Group IIc (light blue) is comprised of one rivet from Turkey and 45 durums, with the majority from Southwest Asia (n = 26) (Figure 3). Group IId (dark blue) includes 26 durums from North Africa (n = 17), Syria (n = 1), Spain (n = 5) and Italy (n = 3) (Figure 3). Table 4 presents these groups' gene diversities. Group Ia had the greatest gene diversity (0.850) whereas Group IId had the least (0.253).

**Table 4:** Genetic diversities in STRUCTURE-defined clusters of tetraploid wheats defined under the *K* = 6 model.

Group	Size	Mean Alleles per Locus	PIC	Gene Diversity
Ia	43	13.9	0.838	0.850
Ib	8	2.3	0.331	0.380
IIa	62	7.4	0.650	0.686
IIb	51	6.3	0.561	0.602
IIc	45	6.4	0.595	0.635
IId	26	2.9	0.226	0.253
Mixed*	9	4.1	0.581	0.632

* Accessions with less than 60% of proportional membership to any of the clusters defined by STRUCTURE were considered admixed.

**Figure 4:**
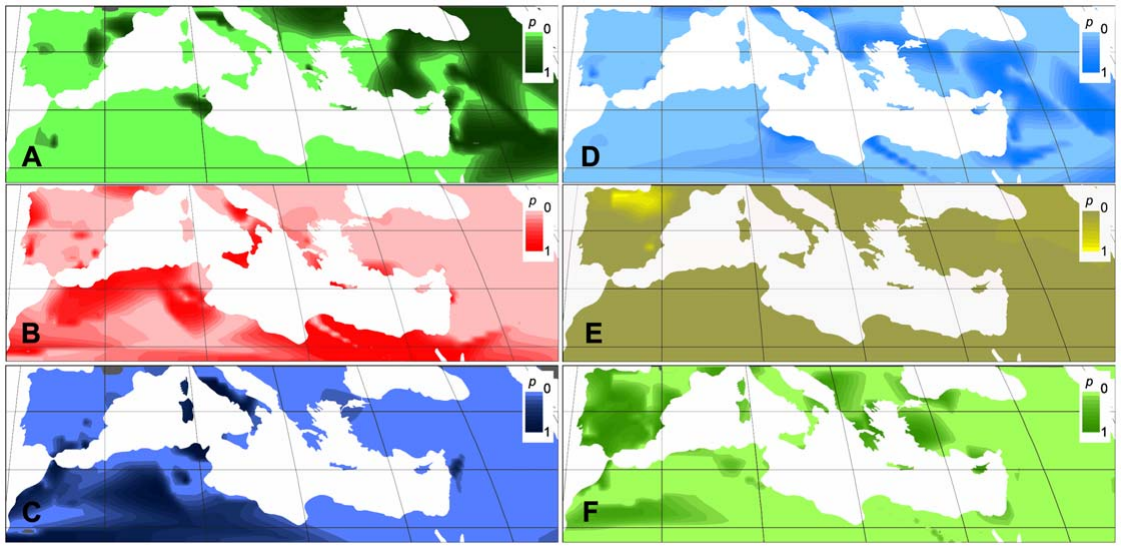
Gene pool frequency clines based on proportional membership of accessions to the six STRUCTURE clusters.

Using *Q*-matrices from the *K = *6 model, we plotted clines of the six groups' allele frequencies on a map of the Mediterranean Basin (Figure 4). Group Ia reflects the provenance of emmer and wild emmer accessions as only these are included in this group. Alleles derived from the group Ib ancestral population were found only in Spain and, less frequently, in northeast Turkey. Group IIa alleles are frequent in the Iberian Peninsula and in Greece and Western Turkey. Group IIb is frequent in the south of Italy and North Africa. Group IId is frequent in the Western Mediterranean, especially Tunisia and Italy, with only one accession in Syria. Contrastingly, group IIc is almost exclusively restricted to the Eastern Mediterranean and particularly abundant in the Near East.

**Figure 5:**
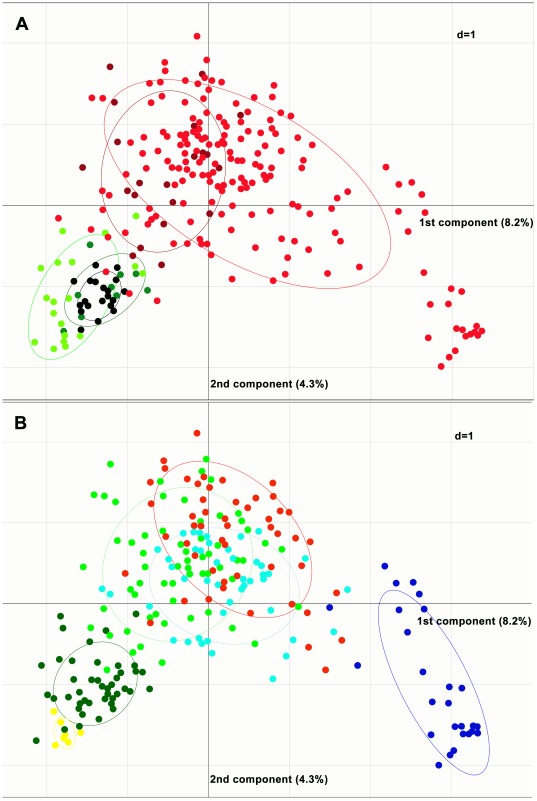
Principle component plots of individual accessions characterised by 29 nuSSRs. In the upper panel, samples were coloured according to form (black: wild emmer; dark green: bread wheat; light green: emmer; dark red: rivet; red: durum). In the lower panel, samples were coloured according to membership to one of the six groups defined by STRUCTURE under the *K* = 6 model.

PCA corroborated the results obtained using STRUCTURE (Figure 5). The first component of the PCA explained 8.2% of the variation and the second component explained 4.3%. The first two components separate emmer, wild emmer and bread wheat from rivet and durum. The group of rivets largely overlaps with that of durum. Accessions corresponding to STRUCTURE groups IId and Ib were notably distinct from the surrounding accessions.

### Chloroplast SSRs

**Figure 6:**
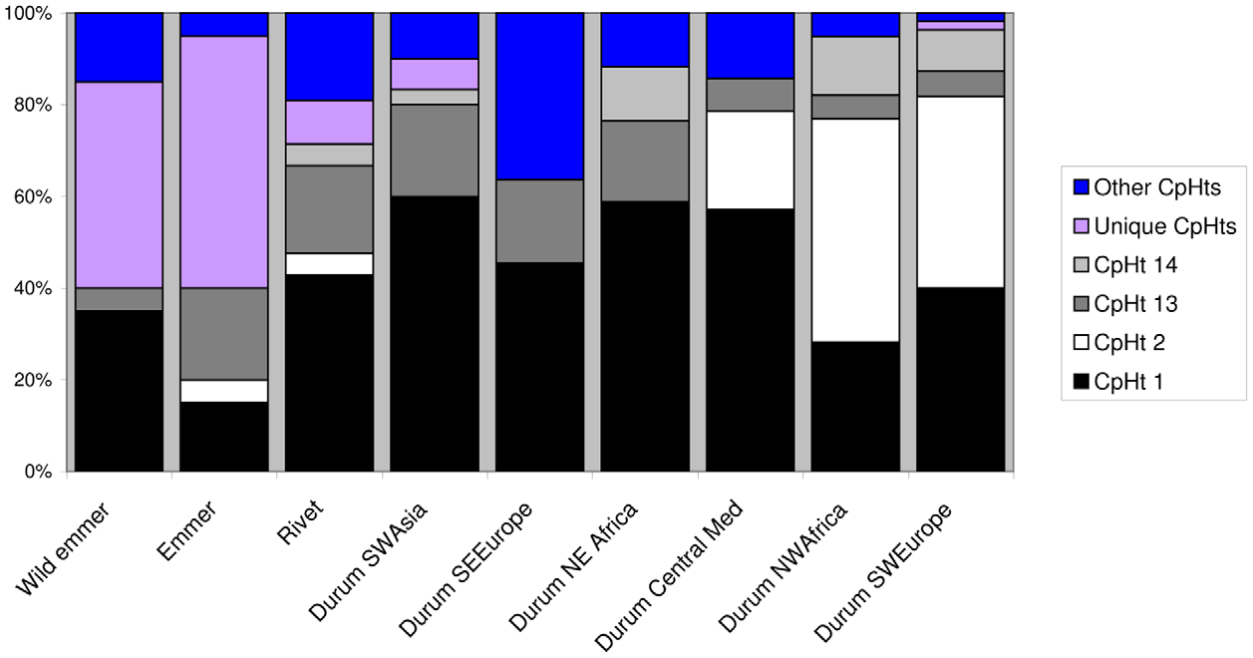
Chloroplast haplotype (CpHt) frequencies in wild emmer, emmer, rivet and durum.

**Figure 7:**
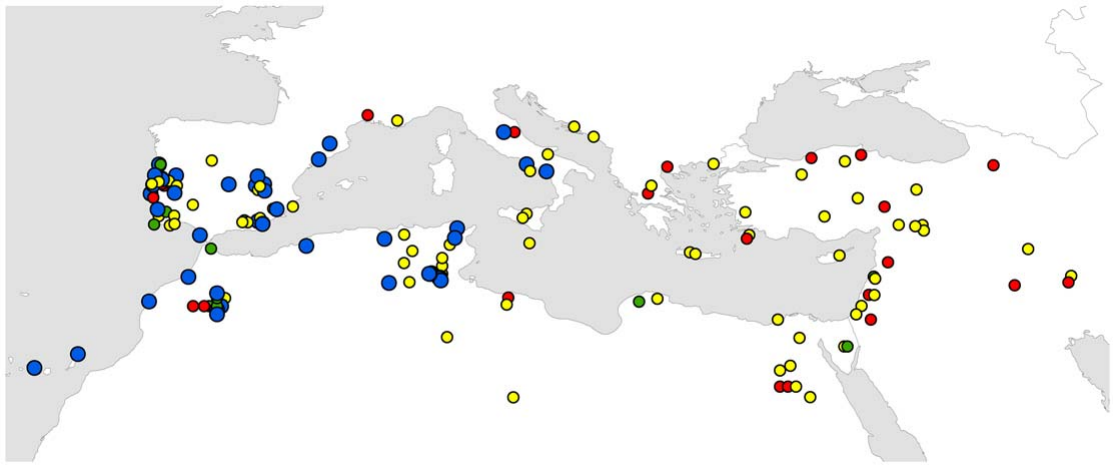
Geographical distribution of the four main chloroplast haplotypes in domesticated tetraploid wheats. cp-haplotype 1 (yellow dots), 2 (blue), 13 (red) and 14 (green).

We identified 24 chloroplast haplotypes (cp-haplotypes) in our accession panel (excluding *T. aestivum*) (Table S5; Figure 6). Cp-haplotypes 1 (which characterised the *T. aestivum* var. Chinese Spring control), 2, 13 and 14 were the most frequent. Emmer and wild emmer had a high frequency of unique cp-haplotypes, indicating a higher diversity of maternal lineages in these forms. The frequencies of the four main cp-haplotypes in Southwest Europe, Northwest Africa and the Central Mediterranean were similar. Cp-haplotype 2 was very rare in emmer and rivet and its geographic distribution in durums was limited to accessions from Northwest Africa, Southwest Europe and Central Mediterranean (Figure 7). 20 durum accessions out of the 26 that were included in nuSSR group IId, also had cp-haplotype 2 in their chloroplast genome. Another 3 accessions in group IId had cp-haplotypes 1, which differs from cp-haplotypes 2 by only one base pair in marker *WCt22* (Table S5).

### ISBP Markers

We found 13 ISBP allelic combinations (Table S6; Table S7). The highest diversity of haplotypes was observed in wild emmer with 10 haplotypes detected. The haplotypes present in rivet were the same that were present in durum. Excluding one Northwest African accession, no haplotype present in the durums and rivets was found in emmer. Interestingly, with the exception of a single accession, all haplotypes detected in emmer, rivets and durums were also present in wild emmer.

### Functional Markers

Almost all of the accessions had neither of the two *Ppd-A1* deletions. The exceptions were five durum landraces CItr 2428 (Egypt), Beladi Bouhi (Egypt) and Rubio o Rubial (Spain) with the ‘GS-105’ type deletion; Rubio (Spain) and Tri13912 (Libya) with the ‘GS-100’ type deletion; and Trigo Fuerte (Spain) where both deletions were detected. The presence of both mutations in this latter accession may be because bulks of 6 to 8 plants were used for DNA extractions and plants with the two types of mutations were included; at a phenotypic level all of the plants are expected to have the same photoperiod insensitivity response. However, these molecular results were not confirmed with the phenotypic analysis of their photoperiod response type. The accessions with the deletion were previously clustered in distinct groups by STRUCTURE.

Diagnostic bands for the presence/absence of a deletion (Langdon-type, as it was originally observed in the “Langdon” variety) in the intron 1 of the *VRN-A1* gene was obtained in 235 of the 244 accessions (Table S1). With only two exceptions (emmer BGE 012302 and wild emmer Tri27996), all the emmer and wild emmer accessions lacked the deletion, whereas in rivets and durums accessions with and without the Langdon-type deletion were observed. Some accessions (19 out of 235) had diagnostic fragments for both the intact sequence and the Langdon-type deletion. This might be due to the presence of more than one genotype per accession in the bulk extracts. Fu and colleagues [33] also reported the presence of both fragments in ten plants within a single accession suggesting that this might be the result of a duplication of the region rather than an issue of heterozygosity.

### Geographic and Environmental Factors

For the 53 durum accessions from the Iberian Peninsula, a statistically significant but weak correlation (*r* = 0.211; *p*<0.001) was found between genetic and geographic distance (Table 5). Further significant but weak correlations were found between genetic and geographic distances in other subsets of accessions tested for isolation-by-distance: in the North African durum samples assigned by STRUCTURE to group IId (*r* = 0.191; *p*<0.001) and in the Mediterranean European accessions (*r* = 0.202; *p*<0.001). For the 53 Iberian durum accessions, there were no noteworthy correlations between *D_C_* and the logarithm of *D_ENVij_* or between *D_C_* and the individual environmental parameters, suggesting that none of them is having a substantial influence on microsatellite genetic diversity (Table 5; Table 6).

**Table 5:** Correlations between geographic, environmental and genetic distances for 53 Iberian durum wheat accessions.

	*D_C_*	*LogD_GEO_*	*LogD_ENV_*
***D_C_***	-	-	**-**
***LogD_GEO_***	0.211*	-	**-**
***LogD_ENV_***	0.009**	0.223*	-

*D_C_*_:_ genetic distance; *LogD_GEO_*_:_ logarithm of geographic distance; *LogD_ENV_*_:_ logarithm of environmental distance.

* Significant (*p*<0.001).

** Non-significant (*p*>0.05).

**Table 6:** Correlation between genetic distances and environmental parameters between landrace localities.

	*D_C_*	r	s	t	u	v	w
***D_C_***	-	-	-	-	-	-	-
**r**	0.094	-	-	-	-	-	-
**s**	0.011	0.224	-	-	-	-	-
**t**	0.053	0.128	0.097**	-	-	-	-
**u**	0.135	0.468	0.420	0.180	-	-	-
**v**	0.139	0.217	0.220	0.401	0.282	-	-
**w**	0.024	0.101	0.452	0.099*	0.418	0.107*	-
**x**	0.161	0.254	0.192	0.363	0.345	0.728	0.117*

Environmental parameter distances are the logarithm of the absolute value of differences between parameters for each accession's location. *r*: Altitude; *s*: mean annual temperature; *t*: maximum temperature of the warmest month; *u*: minimum temperature of the coldest month; *v*: mean annual precipitation; *w*: precipitation of driest month; *x*: precipitation of wettest month.

* Significant (0.001<p<0.05);

** Non-signficant (p>0.05); all other values indicated were significant (p<0.001).

## Discussion

### Taxonomy

Our data support the taxonomical classification of MacKey [17]. Analyses of nuSSRs by PCA, STRUCTURE and NJ dendrograms all separate the hulled tetraploid wheats (emmer and wild emmer) from the naked tetraploid wheats (durum and rivet). Our results agree with those of Li et al [22], Petersen et al [20] and Zhang et al [25] in that durum and rivet share a common gene pool and are indistinguishable genetically. We found no support for rivet and durum being classified as separate taxa using several different marker types (nuSSRs, ISBPs, cp-haplotypes) and several analysis methods (STRUCTURE, PCA, genetic distance approaches).

Maternal cp-haplotype frequencies suggest similarities between rivet and durum from Southwest Asia and Southeast Europe. A similar genetic identity shared by rivets and durums is evidenced by the fact that none of the populations defined by STRUCTURE, modelled using only naked wheats, separates the rivet accessions from the durum ones. Likewise, the clustering of emmer wheat and wild emmer in the same groups in all models considered indicates a broadly shared SSR allele pool, although in this case additional genomic and phenotypic differences related to domestication syndrome traits justify the classification as a different species. Differences in key genes between rivet and durum have not been identified or quantified and it is debatable if the differences in phenotype are sufficient to classify durum and rivet as different taxa. Our data support the classification that considers rivet and durum as varieties of the same taxon. This suggests that rivet landraces constitute an easily transferable source of genetic variation for durum wheat breeding programmes.

### Genetic Diversity and Evolution

Within population genetic diversity in wheat landraces has seldom been investigated [50]. Using even a small number of SSRs, Hagenblad et al [50] observed that germplasm bank and historic accessions of barley, rye, oat and pea landraces consist of a mixture of genotypes. We tested this assumption for tetraploid wheat landraces still in cultivation and held in germplasm banks. Our low within-landrace genetic diversities are expected since selfing is known to reduce the frequency of heterozygous individuals and hence within-population genetic diversity over time [51]. The relatively high heterogeneity observed in Conforcos and Pelugano is probably due to deliberate breed crossing from heterogeneous parents or to mixing of seed lots [52]. It is likely that the fields in these villages were sowed with a mixture of grains from two sources.

Genetic diversity between accessions or within groups of accessions was significantly higher than within accessions. Wild emmer and emmer accessions were more diverse than rivet or any group of durums (Table 2). The clustering by STRUCTURE of wild emmer with emmer agrees with the expectation of domesticated emmer having evolved from a reduced number of localised populations along the distribution range of wild emmer. It also suggests that, although stretches of emmer's genome underwent significant changes during domestication [53]–[54], the gene pool in neutral regions of the two forms may have not diverged significantly.

It has been assumed that naked wheats evolved from emmer in the Eastern Mediterranean area through a process of diversification [5], [8]. Nevertheless, the possibility of one or more domestications from strands of wild emmer has not been tested to date. The separation of naked wheats (durum and rivet) from the hulled (wild emmer and emmer) suggests that naked forms might have originated from a small number of genotypes, of either emmer or wild emmer, sometime during the Neolithic and expanded in number since then. The differences between naked wheats and emmer evidenced in all our genetic marker systems suggest a strong isolation after domestication. This could have happened if, as suggested by Özkan and colleagues [55], durum was selected from an European emmer population in the Mediterranean region, being thus isolated from other emmer and wild emmer populations. Southeastern European durums are genetically closer to emmer and wild emmer than are other durums (Figure 1), possibly corroborating this hypothesis. Alternatively, the genetic distinctiveness of the naked forms in relation to emmer might be due to its agronomic properties [56]. It is likely that throughout history farmers would cultivate hulled wheats separately from naked forms. The different processing requirements of hulled cereals, distinct ripening times and the fact that these tend to grow more successfully on poorer soils than do naked wheats could have led to the latter being sowed more intensively in fertile soils and the hulled kept in more remote fields. This would lead to a strong reproductive isolation that prevented gene flow between naked and hulled forms. Compared to durum, emmer seems to have been a minor crop in the Mediterranean region since the Classic Period and even since the early Neolithic in the Iberia Peninsula [10]. The lower pressure on uniformity and less intensive management practices could account for the higher genetic diversity observed in emmer (Table 2).

Maternal lineages in emmer and wild emmer also seem to be more diversified. The number of unique cp-haplotypes detected in wild emmer (7) was much higher than in emmer (3), durum (3) or rivet (2). Out of the 14 cp-haplotypes found in rivet and durum, 4 were also present in emmer accessions and 3 were present in wild emmer. Of the 7 cp-haplotypes found in emmer, 2 were also found in wild emmer. This suggests a scenario in which all three forms share a common maternal ancestral gene pool that would later become distinct between them due to different population histories. Zohary and Hopf [7] proposed that the naked forms of tetraploid wheats, probably including both durum and rivet, evolved from domesticated hulled emmer in the Fertile Crescent. Our data support this hypothesis. If this event occurred only a small number of times and in a limited geographic area [6], only a small number of cp-haplotypes, from a broader gene pool present in emmer, would appear in the naked durum and rivet as a consequence of a bottleneck effect. Thuillet and colleagues [57] reported a series of bottleneck effects in the population history of durum landraces, detected as decreases in the effective population size, one of these in the transition from emmer to durum wheat. The genetic distinctiveness of emmer is more pronounced in the ISBP system where, with the exception of one durum from the Maghreb, no haplotype was shared between emmer and naked wheat accessions. Haplotypes present in both emmer and in durum accessions were detected in wild emmer. This suggests an alternate scenario in which naked tetraploid wheats could have been domesticated *de novo* from wild emmer strands, acquiring by mutation and human selection the tough-rachis trait independently from emmer, plus the free-threshing trait that is lacking in the latter.

Considering the strong genetic similarity between rivets and durums demonstrated in all our marker systems, it is likely that the two subspecies were originated from a common domesticated ancestor. Adaptation of plants to specific conditions as the species was introduced into Europe could have yielded landrace varieties with distinct morphological characteristics, such as the distinct head form in rivet or the latter's higher tolerance to frost and humidity in comparison with durum. These differences would be maintained by artificial selection giving rise to the agronomically distinct rivet and durum. These selective pressures were apparently not strong enough to create a distinct genetic pool between the two. Another possibility is that the distinction between rivet and durum is simply an artefact based on the criteria used by early botanists, focusing on differences rather than similarities between groups, whereas traditional farmers might have simply thought in terms of varieties with similar agronomic properties, like thresh-ability [58]. In fact, all cultivated tetraploid wheats are inter-fertile with one another [7].

### Phylogeography and History

Population structure was detected within emmer genotypes. Emmer accessions from Asturias were distinct from other emmers. These include accessions preserved both *in situ* and *ex situ*. In some of these Asturias accessions, Leigh and colleagues [26] detected rare cp-haplotypes that were only detected in emmer strands from Turkey. The null alleles detected with marker *Xgwm169* could also be phylogenetically meaningful as they occurred only in Asturias accessions. Emmer landraces in Asturias can constitute relic varieties descending from the earliest cultivated forms that have been preserved from genetic erosion due to geographic isolation in mountainous terrain and small-scale farming [59].

Durum has a more complex phylogeography. Group IId is almost exclusively restricted to the Western Mediterranean (Figure 4) and has very low gene diversity (0.253) compared with the other groups defined in the *K = *6 model. It appears to be associated with cp-haplotype 2 which also shows an exclusive Western Mediterranean distribution (Figure 7). Genetic proximity between durum accessions from the Western Mediterranean is also visible in the similar frequencies of cp-haplotypes in Iberia, Northwest Africa and Central Mediterranean (Figure 6). The distribution of group IId and its reduced genetic diversity could derive from a recent introduction from a few genotypes followed by geographic expansion. For instance, the Romans are known to have had a profound impact in North African agriculture. The classical author Columella [60] mentions the suitability of North Africa for cereal cultivation and Pliny the Elder [61] reports that a measure of wheat sown would yield 150 times in North Africa. In the Imperial Period, the modern Maghreb was known for its cereal fields, being therefore dubbed, alongside Egypt, the “bread-basket of Rome” [62], [63]. The profound reorganisation of agriculture in this region under Roman rule, attested by written and archaeological records alike, involved the introduction of wheat in previously uncultivated lands made possible by Roman irrigation technology [64]. Possibly, this involved the introduction of uniform genotypes throughout new regions and perhaps in previously cultivated areas where the genetic signatures in old varieties could have been over-stamped.

Using genetic distance-based methods based on AFLP and SSR data, Moragues and colleagues [49] suggested that North African durums were genetically more similar to those from Iberia, which they interpreted as the influence of the Arabs and the medieval Islamic agricultural revolution (based on Watson [65]). Watson's contention that the Arabs introduced durum wheat into North Africa has been discredited on historical and archaeological grounds [66]. We also found no evidence for Arab influence in terms of genetic structure in durum landraces. We observed that durums from Northwest Africa cluster more closely with those from the Central Mediterranean than with those from Iberia (Figure 1), although cluster IIb identified by STRUCTURE fits vaguely the area of influence of the medieval Islamic Empire, where the Arabs are known to have introduced new crops and farming techniques.

#### Structure

results suggest the presence of admixture in Mediterranean accessions. Many landraces received alleles from more than a single gene pool (Figure 2). The occurrence of mixed accessions in the different models could be explained by the spread of tetraploid wheats from more than a single ancestral population (polyphyly). Alternatively, gene flow between different varieties might have occurred frequently in the past. This could have happened by means of introduction of new genotypes in fields, followed by genetic exchange of alleles via introgression. Introgression is reported even in predominantly self-pollinated species like wheat. It occurs after hybridization when two species, or ecotypes, meet, as migration events bring the two into proximity [67]. This can be seen as evidence for a dynamic history of wheat farming in the Mediterranean, with introductions of genotypes by means of seed exchange between distinct regions, followed by the formation of admixed varieties and possible extinction of certain genotypes. The presence of distinct genotypes in the fields of Pelugano and Conforcos illustrates how fields tend to be diversified in traditional agricultural practices, creating opportunities for gene flow and increased genetic diversity.

We hypothesised that the phylogeographic pattern observed for tetraploid wheats could be explained either by migration (introduction of varieties) or natural selection (environmental factors affecting the plants adaptation). We investigated variation in our accession panel for two functional markers, one related to photoperiod response and another to vernalisation requirement. Only five accessions had a deletion in the *Ppd-A1* gene and their geographic distribution did not seem to be significant. Because this deletion was only found in durum accessions, it is likely that it appeared in a few durum lines after this form was domesticated and was probably absent in wild emmer and emmer. For the *VRN-A1* gene the presence of the deletion suggests the accession might not require vernalisation and may thus be sowed as a spring type. Interestingly, although both sequences with and without the deletion were observed in rivets and durums, the deletion sequences were almost absent in emmer and wild emmer. Kato et al. [68] studied geographical variation in vernalisation requirements in Near Eastern wild emmer accessions and found both types in most populations, with a predominance of winter types. The authors suggest that the spring type might have evolved from previous winter types as an adaptation to warmer conditions, and predicted that the predominance of spring types in tetraploid wheats may reflect cultivation under warmer temperatures. It is likely that in durum, a crop of warmer Mediterranean conditions, a selective pressure was applied to favour spring types, whereas in emmer, traditionally cultivated in mountainous and inland regions where the vernalisation requirement is of importance, this pressure was absent. In durum both types were present in all regions suggesting that the lineages containing the deletion may have been present from the origin of durum spread.

Weak correlations between geographic and genetic distances suggest that a simple isolation by distance model does not apply to durum landraces in Iberia, North Africa or Mediterranean Europe. It is probable that durum wheat did not follow a linear and gradual route when it was introduced in Iberia, North Africa or Mediterranean Europe, or that subsequent historical processes have masked the primitive phylogeographic signal.

The absence of correlations between genetic distances and environmental parameters for the Iberian durum suggests that natural selection has not significantly affected SSR allele distribution in Iberia. This is not to say that some plants would not have been selected due to their better performance under different environmental conditions, but simply that the genetic signal of this selection was not imprinted in the SSR markers analysed. Gene flow (by human-driven migration of genotypes) seems thus to be the major evolutionary force affecting the phylogeographic pattern of this species as revealed by nuSSR markers.

Our study revealed a distinct population structure in tetraploid wheat accessions using even a small number of SSR markers. It is likely that a larger panel of SSRs would reveal further substructure in these wheat types. It remains to be seen if high-throughput screening of thousands of genome-wide SNPs in these accessions would confirm or falsify the population structure here reported. Moreover, using next-generation-sequencing technologies a large number of homologous genes related to climatic or physiological adaptations could be sequenced in landraces and wild types, revealing selective pressures occurring during wheat adaptation to distinct environments. Although caution would be advised when comparing these different marker systems, namely due to different ascertainment bias levels in SNPs and in SSRs [69], [70], complementary of markers will certainly elucidate finer details in the domestication of wheat and the subsequent emergence of distinct locally-adapted landraces.

### Conclusions

In this paper we have demonstrated that tetraploid wheat accessions in the Mediterranean show population structure that may reflect early agricultural movements. This structure is corroborated by different genetic marker systems and adds strength to the taxonomic classification proposed to tetraploid wheats by MacKey [18]. Wild and domesticated emmer accessions appear to have evolved from a common gene pool, distinct from the gene pool from which rivet and durum originated. Rivet and durum wheats most likely represent varieties of the same taxon with distinct morphological characters. The strong difference between the hulled emmer and the naked rivet and durum suggests that naked wheats evolved from a small number of hulled tetraploid genotypes, and have been in relative reproductive isolation since their spread into Europe and North Africa during the Neolithic. Divisions within the main groups point to a dynamic history of wheat farming in the Mediterranean. This includes a distinct cluster of accessions limited to the Western Mediterranean which are characterised by a very low genetic diversity. Results of within landrace genetic diversity suggest that although wheat accessions tend to be homogeneous, different genotypes may occur, especially in *in situ* preserved varieties. An individual wheat landrace accession cannot always be seen as a more-or-less homogeneous group of individual plants with most alleles being fixed, as landraces are not static entities and episodes of introduction of new genotypes may occur throughout their history.

## Supporting Information

Details S1 **PCR details.**
(DOC)

Figure S1 **Determination of the best STRUCTURE model for the panel of tetraploid wheats.** The most meaningful values of *K* were determined using the *LnP(D)* (blue line) and the *ΔK* (red line) methods.
(TIF)

Figure S2 **STRUCTURE results for domesticated tetraploid wheats (top and middle) and naked wheats (bottom).** For the domesticated tetraploid wheats, models *K* = 3 and *K* = 6 are presented. For the naked wheats, model *K* = 4 is presented Groups were identified with the same letter and colour as their equivalent groups in the STRUCTURE runs using the complete panel of accessions.
(TIF)

Figure S3 **Geographical distribution of population structure in domesticated tetraploid accessions.** Wild emmer is excluded. Pie charts indicate the proportional membership of each landrace to each one of the different groups as determined by STRUCTURE. A) *K* = 3 model. B) *K* = 6 model.
(TIF)

Figure S4 **Plot of** ***ΔK*** **and** ***LnP(D)*** **for 20 STRUCTURE runs with a panel of 215 tetraploid wheat accessions.**
(TIF)

Table S1 **Accession sourcing and genotype data.** NSG-USDA: National Small Grains Research Facility, Idaho, USA; IPK: Institute für Pflanzengenetik und Kulturpflanzenforschung, Gatersleben, Germany; CRF-INIA: Centro de Recursos Fitogenéticos, Madrid, Spain; ENMP-INIA: Estação Nacional de Melhoramento de Plantas, Elvas, Portugal.
(XLS)

Table S2 **Markers used to characterise genetic diversity in 235 tetraploid wheat and 9 bread wheat accessions.** For each marker chromosome location (Chr.), PCR annealing temperature (T_m_), Dye used for labelling primers, number of alleles detected (Allele Richness), gene diversity and polymorphic information content (PIC) are provided. “Touchdown” PCRs are denoted by “t*” under annealing temperature.
(XLS)

Table S3 **Genetic markers used to characterise the** ***within landrace*** **genetic diversity in seven tetraploid wheat accessions.** For each marker measures of genetic diversity are provided. MAF: frequency of the most frequent allele; PIC: polymorphic information content.
(XLS)

Table S4 **Genetic marker systems used in the characterisation of tetraploid wheat landraces.** For each system the percentage of null alleles detected, average frequency of the most frequent allele (MAF), average number of alleles and average gene diversity detected and polymorphic information content (PIC) is presented.
(XLS)

Table S5 **Chloroplast haplotypes identified in tetraploid wheat accessions combining the alleles of five chloroplast SSR markers.**
(XLS)

Table S6 **ISBP haplotypes detected in tetraploid wheat accessions.** The haplotype found in the T. aestivum var. Chinese Spring control was labelled “I”. Numbers correspond to the number of accessions where haplotypes were detected.
(XLS)

Table S7 **Haplotypes identified in tetraploid wheat accessions using four ISBP markers.**
(XLS)
