# Supplementary material for: Tetraploid Wheat Landraces in the Mediterranean Basin: Taxonomy, Evolution and Genetic Diversity
Source: PLoS One. 2012 May 16;7(5):e37063. doi: 10.1371/journal.pone.0037063 (PMC3353906; doi:10.1371/journal.pone.0037063)
Supplement: Details S1 — PCR details. (DOC) [file pone.0037063.s001.doc]

**SUPPORTING INFORMATION**

PCRs for **genomic SSRs** were conducted in 15μL volumes containing 50ng of genomic DNA, 0.2mM of each dNTP, 1x Roche PCR buffer, 15μg of BSA, 0.32 μM of labelled M13 primer, 0.32 μM of reverse primer, 0,08μM of M13-tailed forward primer and 0.6 units of Roche *Taq* polymerase. PCRs were conducted in a AB-Veriti® thermocycler with the following thermal profile: initial denaturing step for 5 min at 94ºC; followed by 30 cycles of 1 min at 94ºC (denaturation), 1 min at the annealing temperature (see Table 2), and 2 min at 72ºC (extension); 8 cycles of 1 min at 94ºC, 1 min at 53ºC, and 2 min at 72ºC; and a final elongation step at 72ºC for 10 min.

PCRs for **EST-SSRs** were conducted in 10μL containing 50ng of genomic DNA, 0.25mM of each dNTP, 1x Roche PCR buffer, 1x Roche GC-Rich buffer, 0.2 μM of each primer and 1 unit of Roche *Taq* polymerase. PCRs were conducted in a GeneAmp® PCR System 9700 thermocycler with the thermal profile: initial denaturing step for 3 min at 94ºC; followed by a touchdown approach of 10 cycles of 50 sec at 94ºC, 50 sec at annealing temperature (decreasing 1ºC in each cycle, from 62º to 52ºC), and 1.25 min at 72ºC; 35 cycles of 50 sec at 94ºC, 50 sec at 52ºC, and 1.25 min at 72ºC; and a final elongation step at 72ºC for 30 min.

PCRs for **chloroplast SSRs** were conducted in 10μL containing 50ng of DNA, 0.4mM of each dNTP, 1x Roche PCR buffer, 5μg of BSA, 0.2 μM of each primer and 0.25 units of Roche *Taq* polymerase. PCRs were conducted in a AB-Veriti® thermocycler with the thermal profile: initial denaturing step for 9 min at 95ºC; followed by 38 cycles of 1 min at 94ºC, 1 min at 56ºC, and 2 min at 72ºC (extension); and a final elongation step at 72ºC for 7 min.

PCRs for the **ISBP markers** were conducted in 10μL reactions containing 50ng of DNA, 0.4mM of each dNTP, 1x Roche PCR buffer, 0.25 μM of each primer and 0.25 units of Roche *Taq* polymerase. PCRs were conducted in a Techne-Touchgene thermocycler with the following touchdown thermal profile: initial denaturing step for 5 min at 94ºC; followed by 8 cycles of 30 sec at 95ºC, 30 sec at 62ºC minus 1ºC each cycle, 30 sec at 72ºC; 30 cycles at 30 sec at 95ºC, 30 sec at 55ºC, 30 sec at 72ºC; and a final elongation step at 72ºC for 7 min.
