# Supplementary figures and images for: Tetraploid Wheat Landraces in the Mediterranean Basin: Taxonomy, Evolution and Genetic Diversity
Source: PLoS One. 2012 May 16;7(5):e37063. doi: 10.1371/journal.pone.0037063 (PMC3353906; doi:10.1371/journal.pone.0037063)

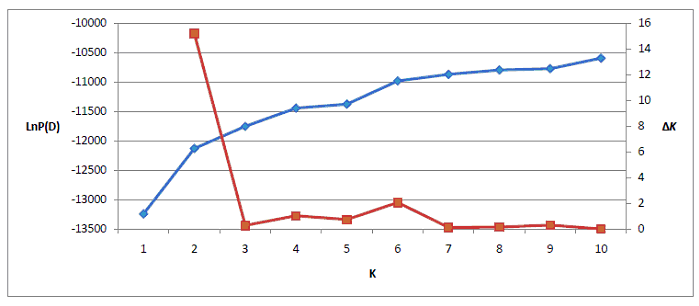

Supplement: Figure S1 — Determination of the best STRUCTURE model for the panel of tetraploid wheats. The most meaningful values of K were determined using the LnP(D) (blue line) and the ΔK (red line) methods. (TIF) [file pone.0037063.s002.tif]

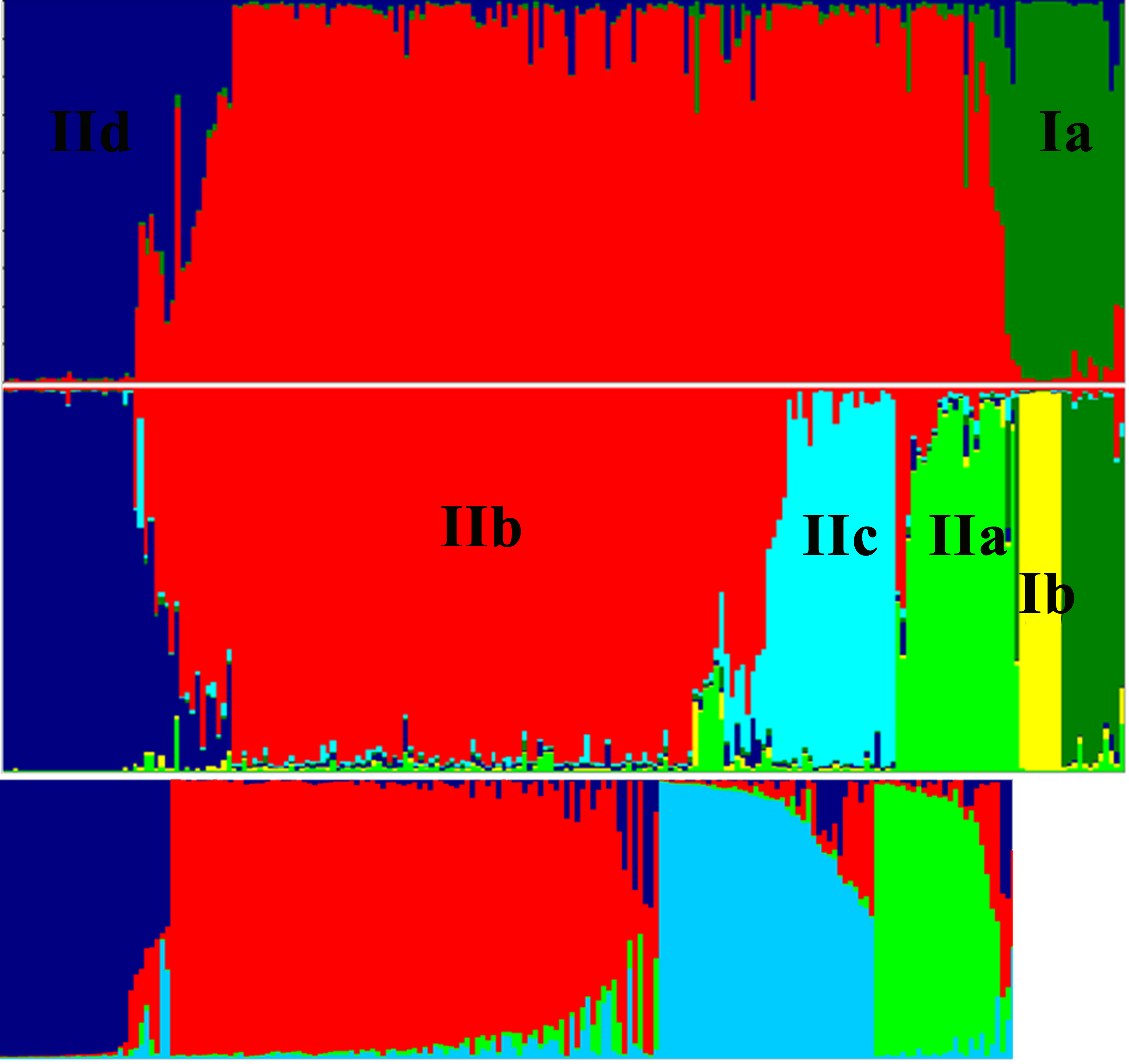

Supplement: Figure S2 — STRUCTURE results for domesticated tetraploid wheats (top and middle) and naked wheats (bottom). For the domesticated tetraploid wheats, models K = 3 and K = 6 are presented. For the naked wheats, model K = 4 is presented Groups were identified with the same letter and colour as their equivalent groups in the STRUCTURE runs using the complete panel of accessions. (TIF) [file pone.0037063.s003.tif]

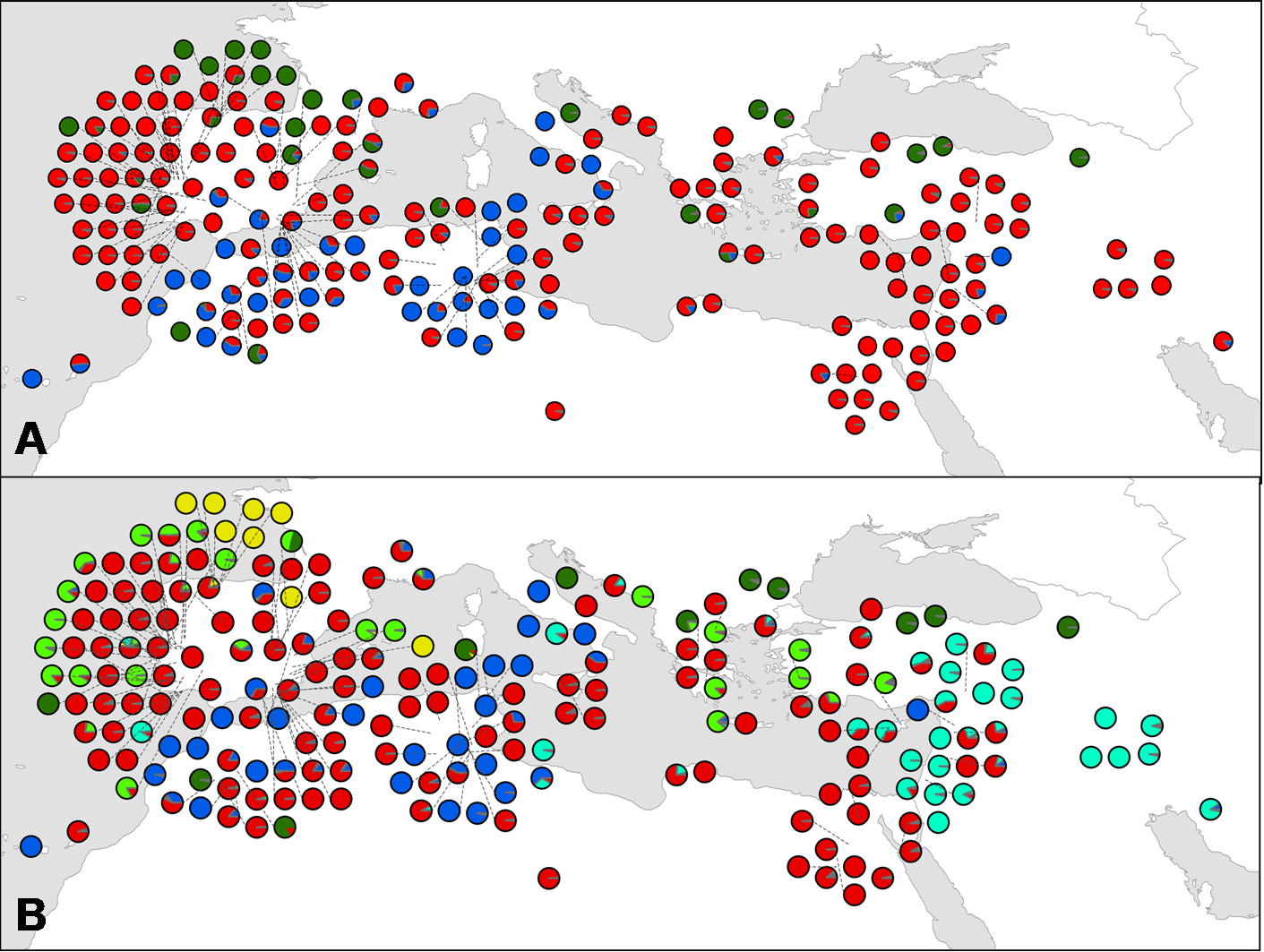

Supplement: Figure S3 — Geographical distribution of population structure in domesticated tetraploid accessions. Wild emmer is excluded. Pie charts indicate the proportional membership of each landrace to each one of the different groups as determined by STRUCTURE. A) K = 3 model. B) K = 6 model. (TIF) [file pone.0037063.s004.tif]

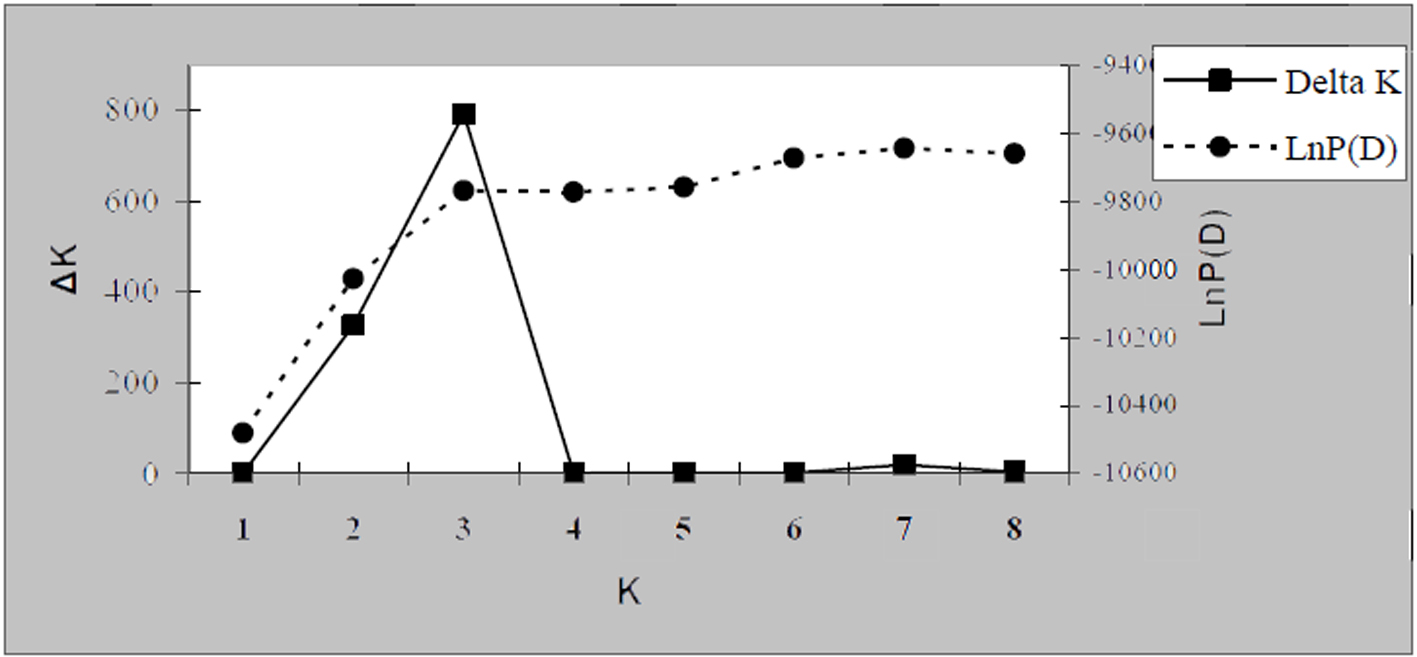

Supplement: Figure S4 — Plot of ΔK and LnP(D) for 20 STRUCTURE runs with a panel of 215 tetraploid wheat accessions. (TIF) [file pone.0037063.s005.tif]
